# Digital Twins in Orthopedics and Trauma: Concepts, Emerging Evidence, and Barriers to Clinical Translation

**DOI:** 10.3390/jcm15114127

**Published:** 2026-05-27

**Authors:** Wojciech Michał Glinkowski, Tomasz Gieroba, Andrzej Śliwczyński

**Affiliations:** 1 Center of Excellence “TeleOrto” for Telediagnostics and Treatment of Disorders and Injuries of the Locomotor System, Department of Medical Informatics and Telemedicine, Medical University of Warsaw, 02-091 Warsaw, Poland; 2 Polish Telemedicine and eHealth Society, 03-728 Warszawa, Poland; 3 Institute of Psychology and Human Sciences, WSEI University, 20-209 Lublin, Polandandrzej.sliwczynski.ahe@gmail.com (A.Ś.)

**Keywords:** digital twins, orthopedics, musculoskeletal modeling, clinical decision support, patient-specific simulation, precision medicine, digital health, biomechanics, rehabilitation monitoring, translational medicine

## Abstract

**Background/Objectives:** Digital twin technology has attracted growing attention in orthopedics for its potential to support patient-specific modeling, simulation, and data-driven clinical decision-making. However, despite the rapid growth in the literature, clinical adoption remains limited, and the term “digital twin” is often applied inconsistently to fundamentally different technological approaches. To establish a clear, function-oriented definition and taxonomy of digital twins in orthopedics, to map current applications across subspecialties, and to critically assess the level of clinical evidence supporting their use. **Methods:** A structured narrative review was conducted using targeted searches of major bibliographic databases (PubMed, Web of Science, Scopus), publisher platforms, and complementary semantic search tools. The retrieved literature was interpreted using a functional analytical framework focusing on patient specificity, data integration, intended clinical role, and degree of clinical validation. Rather than conducting a formal, systematic appraisal, the aim was to provide a concept-driven synthesis of the field and identify patterns of use, maturity, and translational limitations. **Results:** Most reported orthopedic digital twin implementations appear to represent static patient-specific simulations supported primarily by preclinical or feasibility-level evidence. Monitoring-oriented digital twins have been more commonly reported in spine care, rehabilitation, and sports medicine, enabling longitudinal assessments but offering limited predictive or decision-support value. Decision-oriented digital twins are uncommon, yet they seem to be the most clinically mature type described in the current literature; so far, only one randomized controlled trial has demonstrated improved decision quality in arthroplasty care. Fully integrated hybrid or closed-loop digital twins remain largely experimental. **Conclusions:** Digital twin technology in orthopedics is characterized by substantial conceptual heterogeneity and limited clinical validation. Near-term clinical impact is most likely to arise from narrowly focused, decision-oriented, and monitoring-based digital twins, although this projection remains dependent on further clinical validation. Greater definitional clarity, functional transparency, and rigorous clinical evaluation are essential to support meaningful translation into routine orthopedic practice.

## 1. Introduction

**Figure 1:**
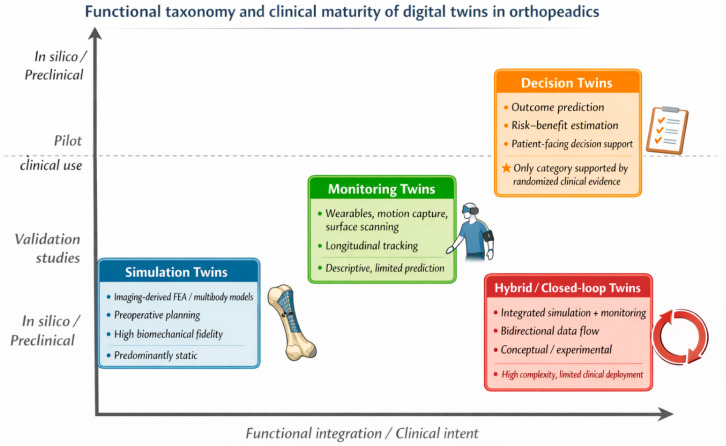
Functional taxonomy and clinical maturity of digital twins in orthopedics. Digital twin applications in orthopedics can be classified into four functional categories: simulation twins, monitoring twins, decision twins, and hybrid or closed-loop systems, which reflect increasing levels of data integration and clinical intent. The horizontal axis represents functional integration and the intended clinical role, and the vertical axis represents clinical evidence and translational maturity. Most orthopedic implementations currently cluster in the simulation and monitoring categories and are supported primarily by preclinical or validation-level evidence. Despite their comparatively lower biomechanical complexity, decision-oriented digital twins are the only category supported by evidence from randomized clinical trials. Hybrid or closed-loop digital twins remain largely conceptual or experimental with limited clinical deployment. The vertical positioning reflects qualitative translational maturity rather than a quantitative comparison between categories. The proposed taxonomy is intended as a functional interpretive framework rather than a formally validated classification system.

Digital twin technology has emerged as a widely discussed paradigm in digital medicine, driven by patient-specific modeling, simulation, and data-driven clinical decision support [1,2,3]. In orthopedics and musculoskeletal care, these promises are compelling, given the clinical challenges posed by anatomical variability, biomechanical complexity, and heterogeneous treatment outcomes [4]. The term digital twin has been rapidly adopted in orthopedic research, spanning applications from preoperative biomechanical simulations to artificial intelligence-enabled tools for outcome prediction [4,5,6]. Despite this expansion, the clinical penetration of digital twins in orthopedics remains limited. At the same time, hundreds of publications reference digital twin concepts, but few report clinical validation or demonstrate improved patient outcomes [7,8]. This gap reflects technical and infrastructural constraints, as well as a lack of conceptual clarity [3,7,9]. Many studies describe static patient-specific computational models as digital twins, despite their lack of key features such as longitudinal updating, clinical workflow integration, or explicit decision-support functionality [7,8,10]. Orthopedics presents a demanding test case for digital twin technology [4,6]. Musculoskeletal systems are load-dependent and exhibit nonlinear biomechanical behavior, whereas clinical outcomes often weakly correlate with static anatomical measures [11,12]. Unlike cardiovascular [13] or oncological [14] applications, where imaging biomarkers may reflect disease severity or treatment response, orthopedic outcomes depend on functional performance, patient-reported symptoms, and dynamic interactions among implants, soft tissues, and neuromuscular control [12,14]. These factors complicate digital modeling and clinical validation, which helps explain why many sophisticated orthopedic digital twins remain in preclinical or experimental settings [4,6]. The proliferation of heterogeneous “digital twin” claims has created a fragmented literature in which technological sophistication, clinical readiness, and evidentiary strength are often conflated [3,7,8,10]. Without a functional taxonomy and a more disciplined appraisal of current implementations, expectations regarding clinical translation may become unrealistic, potentially undermining confidence in promising applications [3,7,10]. This review aims to (1) define and classify digital twins in orthopedics, (2) map digital twin applications across orthopedic subspecialties and clinical evidence levels, and (3) examine barriers to routine clinical adoption. By distinguishing ambition from clinical value, this review provides a framework for future research, validation, and implementation of digital twin technology in orthopedic care. To clarify functional heterogeneity and translational maturity, a functional taxonomy was proposed (Figure 1).

### 1.1. What Is (And Is Not) a Digital Twin in Orthopedics

The term digital twin has been increasingly adopted in orthopedic and musculoskeletal research; however, its use remains conceptually heterogeneous and frequently imprecise [4,6,8]. In many publications, patient-specific computational models—particularly finite-element or multibody simulations derived from medical imaging—are labeled as digital twins despite lacking key defining characteristics [6,15]. This definitional ambiguity risks obscuring both the true potential of digital twin technology and the reasons for its limited clinical translation [3,7,10].

### 1.2. Core Definition

In the context of orthopedics, a digital twin should be defined as a dynamic, patient-specific digital representation of an orthopedic system that integrates anatomical, biomechanical, and clinical data and is designed to inform clinical decision-making through simulation, prediction, or monitoring, with the capacity to be updated as new data become available [7,10,16]. This definition builds on recent conceptual frameworks in digital medicine while emphasizing features that are particularly relevant and challenging in musculoskeletal care. Crucially, patient specificity alone is insufficient to qualify a system as an ideal digital twin. Rather, digital twins exist on a continuum of functional maturity, distinguished by their levels of dynamism, data integration, and clinical purpose [7,10,16].

### 1.3. Distinguishing Digital Twins from Patient-Specific Models

A large proportion of orthopedic studies described as digital twin applications rely on static, one-time computational models, most commonly finite element simulations generated from preoperative CT or MRI. Although these models may be highly accurate representations of patient anatomy or joint mechanics, they typically lack mechanisms for data updating, interaction with the clinical environment, or longitudinal use. Such models are better classified as patient-specific simulations rather than as digital twins. Conflating these concepts inflates perceptions of technological maturity and creates unrealistic expectations regarding clinical readiness. This distinction is particularly important in orthopedics, where the biomechanical behavior is load-dependent, context-sensitive, and often nonlinear [6,10,17].

### 1.4. Functional Taxonomy of Digital Twins in Orthopedics

Orthopedic digital twins can be categorized into four functional types based on their primary function and level of integration, thereby clarifying the landscape [4,18,19,20] (Figure 1). The proposed functional classification was developed through iterative interpretation of the relevant retrieved literature by the authors, focusing on differences in data integration, longitudinal updating, intended clinical role, and degree of clinical implementation. The taxonomy is intended as a pragmatic interpretive framework rather than a formally validated classification system.

#### 1.4.1. Simulation Twins

Simulation twins are the most commonly reported form in the literature. These systems use patient-specific anatomical data, typically from imaging, to simulate biomechanical behavior under predefined conditions, such as joint loading, implant alignment, and fracture fixation strategies [6,15,16,21,22]. Simulation twins are typically characterized as static or quasi-static models designed to address specific clinical questions and are not updated after their initial development [10]. They are well-suited for preoperative planning and hypothesis testing, particularly in arthroplasty and trauma, but do not constitute full digital twins in the strict sense of the term. Most orthopedic “digital twin” publications currently fall into this category [6,15,16].

#### 1.4.2. Monitoring Twins

Monitoring twins involves longitudinal data streams, such as wearable sensors, motion capture, or repeated imaging, which enable the evolution of digital representation [6,23]. These systems have been most frequently explored for spine deformity monitoring, rehabilitation, and sports medicine. Their defining characteristics include repeated or continuous data acquisition, temporal tracking of functional or anatomical changes, and limited or absent predictive capability. Monitoring twins move closer to the digital twin paradigm by enabling dynamic updates; however, they often remain descriptive rather than decision-supportive [23,24,25,26].

#### 1.4.3. Decision Twins

Decision twins (decision-support-oriented digital twins) are explicitly designed to support clinical decision-making, often by integrating patient-reported outcomes, clinical variables, and predictive models [27,28,29,30]. In orthopedics, this category includes AI-enabled systems that estimate the risks, benefits, or expected outcomes of interventions such as joint replacement [28,29,30,31]. Unlike simulation twins, decision twins may not rely heavily on biomechanical modeling; their value lies in personalized prediction rather than physical fidelity. Decision twins are the only class supported by randomized clinical trial evidence in orthopedic care, underscoring their relative maturity from a translational perspective [4,15,32].

#### 1.4.4. Hybrid or Closed-Loop Twins

Hybrid digital twins combine elements of simulation, monitoring, and decision support with the theoretical capability for bidirectional interaction between the patient and digital model. These systems may integrate imaging, biomechanical simulation, sensor data, and clinical feedback within a single framework [1,2,4,8,33,34]. In orthopedics, such closed-loop twins remain largely conceptual or experimental. Substantial computational demands, data integration challenges, and the absence of validated verification and uncertainty quantification frameworks constrain their realization [4,6,35].

### 1.5. Implications for Clinical Translation

This taxonomy highlights a central paradox in orthopedic digital twin research: the most technically sophisticated systems are often the least clinically mature. Simultaneously, most clinically validated implementations rely on comparatively simple models [2,4,18,31]. The widespread mislabeling of static simulations as digital twins further obscures this fact. Therefore, a clear functional classification is essential, not only for accurate reporting but also for aligning expectations regarding clinical readiness, regulatory pathways, and future research priorities. Without such clarity, the term digital twin risks becoming a catch-all descriptor that undermines its translational value [16,36,37,38].

## 2. Materials and Methods

This study was designed as a structured narrative review with a concept-driven analytical framework rather than a formal systematic review. The primary objective was to clarify definitions, organize the heterogeneous literature, and critically interpret the translational status of digital twin applications in orthopedics.

### 2.1. Literature Identification and Search Strategy

Relevant literature was identified through targeted searches of major bibliographic databases, including PubMed, Web of Science, and Scopus, supplemented by searches of publisher platforms (e.g., Springer Nature, ScienceDirect, BMJ Journals, Taylor & Francis) and semantic search tools such as Google Scholar and Elicit. Search terms combined keywords related to digital twin technology and musculoskeletal care, including “digital twin”, “orthopedics”, “musculoskeletal”, “arthroplasty”, “spine”, “trauma”, “rehabilitation”, “biomechanics”, and “patient-specific modeling”. Additional relevant publications were identified through citation tracking of key reviews and seminal articles.

The search strategy was designed to capture conceptually relevant literature rather than to provide an exhaustive or fully reproducible dataset, in line with the interpretive aims of this review.

The literature search yielded 111 unique records after deduplication, of which 73 were included after title/abstract screening and full-text review (Supplementary Table S1). Study counts should be interpreted as indicative rather than exhaustive.

### 2.2. Eligibility and Selection Approach

Publications were considered relevant if they described digital twin concepts or closely related patient-specific digital modeling approaches in clinically meaningful orthopedic or musculoskeletal contexts, including simulation, monitoring, prediction, or decision support. The selection process was based on expert assessment of relevance and conceptual contribution rather than formal inclusion/exclusion workflows typical of systematic reviews. Studies lacking clinical context or functional relevance were excluded.

### 2.3. Analytical Framework

The included literature was interpreted using a functional framework examining: patient specificity, degree of data integration, presence of longitudinal updating, intended clinical role (simulation, monitoring, decision support), and level of reported clinical validation. Based on these dimensions, applications were organized into four functional categories: simulation twins, monitoring twins, decision twins, and hybrid/closed-loop systems.

### 2.4. Assessment of Clinical Maturity

Clinical maturity was interpreted pragmatically based on study design, validation setting, and degree of clinical implementation. No formal risk-of-bias tool or quantitative evidence grading system was applied due to substantial heterogeneity in study types and objectives.

### 2.5. Scope and Limitations

This review prioritizes conceptual clarity and translational interpretation over exhaustive evidence synthesis. As such, the findings should be understood as an interpretive mapping of the field rather than a formal systematic evaluation of all available studies. This review focuses on conceptual clarity, clinical relevance, and translational readiness, rather than an exhaustive enumeration of all published digital modeling studies. Quantitative synthesis was not performed because the primary objective was to critically interpret the current state of the field and identify the structural barriers to clinical adoption. This approach is consistent with the review’s emphasis on distinguishing true digital twin implementations from advanced, but static, patient-specific models. Many publications that used digital modeling approaches were intentionally excluded because they did not meet the functional criteria for a digital twin as defined in this review. An extended overview of published orthopedic digital twin studies is provided in Supplementary Table S2.

## 3. Results

### 3.1. Current Landscape of Digital Twin Applications in Orthopedics

Across orthopedic subspecialties, most reported digital twin applications remain preclinical, focusing on static patient-specific simulations, whereas clinically validated implementations are uncommon. Studies varied widely in their intended clinical roles, data integration, and validation levels, highlighting the substantial heterogeneity in current digital twin implementations. The functional taxonomy and clinical maturity of digital twin applications across orthopedic subspecialties are summarized in Table 1.

### 3.2. Arthroplasty and Joint Preservation

Digital twin applications in arthroplasty are dominated by simulation-based models from patient-specific imaging used for preoperative biomechanical analysis. These systems are static and narrowly scoped, addressing single questions such as implant alignment, joint mechanics, cartilage loading, and patellofemoral tracking [4,6,40]. Arthroplasty is the most-studied domain in orthopedic digital twins, with research primarily focused on the knee joint and fewer studies on the hip, shoulder, or ankle [4,6,47]. Some studies have incorporated monitoring components, including sensor-based measurements of joint loading or alignment after implantation. Although these approaches enable longitudinal tracking, they lack predictive or decision-support functionality. Decision-oriented digital twins are rare but appear to be the most clinically proximate category in the current literature [6,48]. The apparent greater maturity of decision twins should be interpreted with caution, given the limited number of outcome-based studies. Only one randomized clinical trial has evaluated a digital twin-based decision-support tool for knee arthroplasty, demonstrating improved decision quality compared with education alone [31]. This study remains the sole source of randomized clinical outcome evidence for DT-informed orthopedic care. Despite technical maturity in anatomical reconstruction and biomechanical simulation, most arthroplasty digital twins remain in preclinical evaluation or pilot clinical use [4,6].

### 3.3. Spine

Spine-related digital twin applications were distributed more evenly across the simulation and monitoring categories than in other orthopedic subspecialties. Simulation twins have been used to model spinal biomechanics, deformity correction strategies, and load distribution across various postural and surgical scenarios [18,49]. These models are derived from imaging data and motion capture and validated against experimental or computational benchmarks rather than clinical outcomes [4,6,18]. Monitoring-oriented digital twins are prevalent in spinal care. Studies have described systems for the longitudinal assessment of spinal deformities, posture, or functional movements using surface scanning, wearable sensors, or repeated measurements. These approaches enable dynamic updates to digital representations and are often embedded in telemedicine or remote monitoring frameworks [6,43,50]. Despite the maturity of longitudinal assessments, most spine-related digital twins remain descriptive rather than predictive, with limited integration into clinical decision-making pathways [1,7,27].

### 3.4. Orthopedic Trauma

Trauma-related digital twin applications are predominantly simulation-based and focus on patient-specific fracture mechanics and fixation strategies. These systems are used for retrospective analysis or preoperative planning in complex cases and require extended processing times, which are incompatible with acute care workflows [42,51]. The clinical implementation of trauma digital twins is uncommon, and monitoring or decision-support components are largely absent. No trauma-related digital twin systems have been evaluated in randomized or prospective clinical trials to date. Despite their potential to support personalized fixation strategies and postoperative management, trauma digital twins remain experimental [15,18,27,32].

### 3.5. Sports Medicine and Rehabilitation

Digital twin applications in sports medicine and rehabilitation commonly incorporate monitoring components that combine biomechanical modeling with wearable sensors, motion capture, or electromyography to track performance, rehabilitation progress, and recovery [25,52]. Simulation elements typically model joint loading or tissue stress during sports-specific movements [6,52]. Monitoring-oriented digital twins enable dynamic updates to digital representations, reflecting changes in movement patterns and neuromuscular control during rehabilitation. Studies have proposed hybrid frameworks that integrate simulation and monitoring components; however, these approaches remain conceptual. Decision-oriented digital twins are uncommon, and clinical validation is limited to feasibility or accuracy studies rather than to outcome-based evaluations [6,25,53].

### 3.6. Distribution of Evidence and Maturity Levels

Most orthopedic digital twin implementations are supported by preclinical evidence, including in silico simulations, experimental validation, and small case series. Studies assessing measurement accuracy or model fidelity are more common than those evaluating clinical impacts [2,7,27]. Only one randomized clinical trial has been conducted, and few studies have reported prospective clinical deployment beyond pilot settings. While this single trial should not be interpreted as definitive evidence, it currently represents the highest level of clinical validation available in this field. Hybrid or closed-loop digital twins that combine simulation, monitoring, and decision support remain theoretical, with no large-scale clinical validation reported [4,31]. These observations suggest that, while digital twin concepts are widely explored in orthopedics, clinical maturity varies across application types, with decision-oriented implementations showing the strongest evidence despite having simpler underlying models [2,6,8,27,31]. Clinical evidence supporting orthopedic DT applications remains limited and heterogeneous, with only one randomized trial and a few validation studies identified (Table 2).

## 4. Discussion

This review highlights the discrepancy between the conceptual promise of digital twin technology and its clinical application in orthopedics. Although publications on digital twins have increased rapidly, most implementations remain preclinical, static, and narrow. Only a few have progressed to clinical integration, and even fewer have demonstrated patient care benefits.

### 4.1. Conceptual Inflation and Definitional Drift

A striking observation from this review is the extent of conceptual inflation surrounding the digital twin. In orthopedic research, static patient-specific simulations—often technically sophisticated and biomechanically detailed—are labeled as digital twins, despite lacking longitudinal updating, interaction with clinical workflows, or decision-support functionality. Thus, fundamentally different technologies were lumped under a single label [4,7,8]. This definitional drift is not merely semantic. Overstating technological maturity creates unrealistic expectations regarding clinical readiness and may erode clinician confidence when the benefits fail to materialize. A functional taxonomy, as proposed in this review, is essential for restoring clarity, supporting transparent reporting, and enabling appropriate regulation and rational prioritization of future research efforts [2,7,27].

### 4.2. The Paradox of Sophistication Versus Clinical Value

Across orthopedic subspecialties, a paradox emerges: the most complex digital twins are often the least clinically mature, whereas the most clinically validated ones use simpler models. High-fidelity finite element-based simulation twins, which are capable of detailed stress and strain analyses, remain confined to preclinical or retrospective applications. Computational demands, long processing times, and poor scalability in clinical workflows limit their translation into practice [4,18,40]. In contrast, decision-oriented digital twins, such as AI-enabled tools that support shared decision-making in arthroplasty, have demonstrated measurable clinical benefits despite relying on less-detailed biomechanical representations [4,6,31]. This apparent advantage should nevertheless be interpreted with caution, as it is currently supported by only a very limited number of outcome-based studies. This contrast underscores a critical insight for translational digital medicine: clinical value does not increase linearly with model complexity. In orthopedics, decision support based on clinically meaningful predictors may outperform high-fidelity simulations that are impractical to deploy at scale [31,54].

### 4.3. Orthopedics Is a Uniquely Challenging Field

Orthopedic care presents distinct challenges for implementing digital twins. Musculoskeletal systems are inherently load-dependent and exhibit nonlinear behavior, whereas clinical outcomes correlate only weakly with static anatomical measures [17,55,56,57,58]. Pain, function, and quality of life—the outcomes that matter most to patients—are shaped by neuromuscular control, rehabilitation adherence, psychosocial factors, and patient expectations [59,60,61]. These characteristics help explain why orthopedic DTs have progressed more slowly toward routine clinical use than similar technologies in domains where disease processes are more directly reflected by imaging or physiological biomarkers. They also highlighted the limitations of anatomy-centric digital twins and reinforced the need for models that integrate functional and patient-reported outcomes with structural data [2,4,6,7].

### 4.4. Barriers to Clinical Translation

Several interrelated barriers continue to constrain clinical translation. From a technical perspective, the computational burden of high-fidelity simulations remains incompatible with time-sensitive clinical contexts, particularly in trauma care [15,18]. Methodologically, the lack of standardized frameworks for verification, validation, and uncertainty quantification limits confidence in model outputs and complicates regulatory assessments [22,62]. Clinically, integration with existing workflows, electronic health records, and reimbursement structures remains underdeveloped [3,7]. Data governance and regulatory considerations further complicate deployment [7,22,63,64]. Similar regulatory uncertainties apply in other jurisdictions, including evolving FDA frameworks for adaptive software-as-a-medical-device systems. Orthopedic digital twins often rely on detailed imaging and motion data, raising privacy concerns under existing data protection frameworks [2,3,47,65,66,67,68]. Moreover, regulatory pathways for adaptive and continuously updating digital systems are still evolving, creating uncertainty for developers and health care providers [62,69,70,71].

### 4.5. Pathways Toward Meaningful Progress

Despite these challenges, several pathways offer realistic and near-term clinical potential. Decision-oriented digital twins, particularly those integrating patient-reported outcomes, clinical variables, and imaging-derived features, appear to be the most immediately translatable [4,6,31,66,67,68,72]. Monitoring twins using wearable sensors and remote assessment technologies may further support longitudinal management in spine care, rehabilitation, and sports medicine [4,25,43]. Technological advances are likely to play an enabling role, including automated model-generation pipelines, AI-based surrogate models that reduce computational costs, and tighter integration of functional and patient-reported data [4,6,33,34,73,74,75,76]. Importantly, future progress will depend less on expanding the scope of digital twin platforms and more on narrow, well-validated use cases aligned with specific clinical decisions.

### 4.6. Limitations

This review has several limitations. First, the narrative and interpretive design does not provide a fully reproducible selection process or formal risk-of-bias assessment. Second, the proposed taxonomy has not been externally validated and should be understood as a conceptual framework rather than a definitive classification system. Third, the available clinical evidence remains sparse and unevenly distributed across application types. These limitations should be considered when interpreting the translational implications of the findings.

## 5. Conclusions

Digital twin technology holds promise for advancing personalized orthopedic care; however, its clinical impact remains limited. This review demonstrates that despite the rapidly growing interest, most orthopedic digital twin applications remain confined to preclinical or feasibility settings, with only a small number achieving meaningful clinical validation. The widespread use of the term ‘digital twin’ to describe static patient-specific simulations has contributed to conceptual ambiguity and, in some cases, inflated expectations for clinical application. A function-oriented taxonomy provides a pragmatic framework for distinguishing between simulation, monitoring, decision, and hybrid digital twins, clarifying their intended clinical roles and translational maturity levels. Viewed through this lens, the evidence suggests that near-term clinical value is most likely to emerge not from increasingly sophisticated biomechanical simulations but from focused, decision-oriented, and monitoring-based digital twins aligned with specific clinical questions and workflows. For orthopedics, successful translation depends on prioritizing clinical relevance over model complexity, integrating patient-reported and functional outcomes with anatomical data, and applying rigorous standards for validation and reporting. As regulatory frameworks and computational tools continue to evolve, digital twins should be evaluated not by their technical ambition but by their ability to support better decisions, improve patient experience, and demonstrably enhance clinical outcomes. These conclusions should be interpreted in the context of heterogeneous and still limited clinical evidence. Future research should focus on prospective validation, standardized reporting frameworks, and clearer operational definitions to support the clinical integration of digital twin technologies.

## Figures and Tables

**Table 1:** Functional taxonomy and clinical maturity of digital twin applications in orthopedics.

Orthopedic Domain	Digital Twin Category	Core Data Inputs	Primary Clinical Function	Evidence Level	Representative Studies
Arthroplasty (knee, hip)	Simulation twin	CT/MRI, finite element, or multibody models	Preoperative biomechanical analysis, implant alignment	Preclinical/in silico	Aubert et al., 2021 [15]; Montgomery et al., 2023 [39]; Michaud et al., 2024 [40]
Arthroplasty (knee OA, TKA)	Decision twin	Clinical variables, PROMs, imaging features, AI models	Shared decision-making, outcome, and risk prediction	Randomized clinical trial	Jayakumar et al., 2025 [31]
Spine (deformity, biomechanics)	Simulation twin	Imaging, motion capture, and biomechanical modeling	Load distribution, deformity correction planning	Preclinical	He et al., 2021 [41]; Lomax et al., 2024 [42]
Spine (scoliosis, posture)	Monitoring twin	Surface scanning, wearables, repeated measurements	Longitudinal monitoring, telemedicine follow-up	Validation studies	Suresh et al., 2023 [23]; Suresh et al., 2025 [43]
Trauma (fracture fixation, non-union)	Simulation twin	CT, finite element analysis	Fixation strategy evaluation, stress analysis	Preclinical/case series	Aubert et al., 2021 [15]; Andres et al., 2025 [18]
Sports medicine/rehabilitation	Monitoring twin	Wearable sensors, EMG, motion capture	Functional tracking, rehabilitation monitoring	Feasibility/validation	Di Matteo et al., 2024 [44]; Frossard et al., 2022 [45]
Multidomain (experimental)	Hybrid/closed-loop twin	Imaging, sensors, simulation, AI	Integrated simulation and monitoring	Conceptual/experimental	Quinn et al., 2023 [46]

Most publications describe preclinical or early-stage systems, with few reporting clinical validation and only one randomized clinical trial evaluating a digital twin-enabled intervention [2,7,27,31]. Across orthopedic subspecialties, applications fall into four categories: simulation twins, monitoring twins, decision twins, and hybrid systems, reflecting increasing data integration and clinical intent [6,18,27].

**Table 2:** Clinical evidence supporting digital twin applications in orthopedics.

Study	Orthopedic Domain	Digital Twin Category	Study Design	Sample Size	Primary Outcome	Key Findings
Jayakumar et al., 2025 [31]	Arthroplasty (knee OA, TKA)	Decision twin	Randomized controlled trial	n = 201	Decision quality (K-DQI)	AI-enabled digital twin decision aid significantly improved decision quality compared with education alone
Suresh et al., 2023 [23]	Spine (scoliosis)	Monitoring twin	Validation study	n ≈ 150 scans	Measurement reliability (ATR)	Excellent intra- and inter-observer reliability (ICC > 0.95) compared with the analog scoliometer
Suresh et al., 2025 [43]	Spine (pediatric scoliosis)	Monitoring twin	Validation study	Multiple cohorts	Agreement with clinical reference	High correlation and diagnostic accuracy in telemedicine scoliosis monitoring
Hoyer et al., 2025 [47]	Arthroplasty/OA	Simulation-derived digital twin	Cross-sectional cohort analysis	n > 4000	OA progression and knee replacement risk	Imaging-based digital twin biomarkers associated with OA severity and risk of knee replacement
Andres et al., 2025 [18]	Trauma (fracture non-union)	Simulation twin	Case series	n = 5	Biomechanical stress reduction	Patient-specific digital twin modeling guided revision strategy with reduced implant stress
Di Matteo et al., 2024 [44]	Rehabilitation (hand)	Monitoring/hybrid twin	Feasibility & validation	n ≈ 20	Functional tracking accuracy	Digital twin-based monitoring is feasible for individualized rehabilitation assessments.

## Data Availability

No new data were created.

## Supplementary Materials

The following supporting information can be downloaded at: https://www.mdpi.com/article/10.3390/jcm15114127/s1. Table S1: Source-specific record identification and screening overview; Table S2: An extended overview of representative digital twin applications in orthopedics and musculoskeletal care.
