# Supplementary material for: Digital Twins in Orthopedics and Trauma: Concepts, Emerging Evidence, and Barriers to Clinical Translation
Source: J Clin Med. 2026 May 27;15(11):4127. doi: 10.3390/jcm15114127 (PMC13257615; doi:10.3390/jcm15114127)
Supplement: Supplementary file 1 [file jcm-15-04127-s001.zip › jcm-4301602-supplementary.pdf]

Supplementary Table S1. Source-specific record identification and screening overview

Source	Records retrieved or screened from the source
PubMed	No unique records identified
PubMed Central (PMC)	20
Web of Science	4
Scopus	No unique records identified
ScienceDirect	19
Springer Nature Link	29
BMJ Journals	8
Taylor & Francis Online	1
Google Scholar*	248
Elicit*	111
Semantic Scholar	No additional unique records identified
OpenAlex	No additional unique records identified
Total unique records before screening	111
Included after title/abstract screening and full-text review	73

Footnote

Google Scholar and Elicit were used as complementary semantic search tools. Records retrieved from these platforms were screened for conceptual relevance and deduplicated against bibliographic databases and publisher-platform records. Therefore, source-specific yields are not additive, and only unique records contributed to the total count shown.

Supplementary Table S2. An extended overview of representative digital twin applications in orthopedics and musculoskeletal care.

This table provides an extended overview of representative studies describing digital twins or closely related patient-specific digital modeling approaches in orthopedics and musculoskeletal care.

Study	Year	Orthopedic domain	Anatomical focus	Digital twin category	Core data inputs	Modeling approach	Intended clinical use	Evidence level
Aubert et al.	2021	Trauma	Tibial plateau	Simulation twin	CT	Finite element analysis	Fixation strategy assessment	In silico / case study
Hernigou et al.	2021	Arthroplasty	Ankle	Simulation twin	CT, motion data	ML-assisted biomechanical modeling	Personalized motion axis identification	Preclinical
He et al.	2021	Spine	Lumbar spine	Simulation twin	Imaging, kinematics	Shape-performance DT model	Biomechanical prediction	Preclinical
Montgomery et al.	2023	Arthroplasty	Knee	Simulation twin	Imaging, kinematics	Virtual joint motion simulator	Alignment analysis	Preclinical
Michaud et al.	2024	Arthroplasty	Patellofemoral joint	Simulation twin	Imaging	Multibody dynamics	Tracking prediction	Preclinical
Gatti et al.	2024	Arthroplasty / OA	Knee	Simulation twin	MRI	Neural shape modeling	Cartilage pressure estimation	Preclinical
Jayakumar et al.	2025	Arthroplasty	Knee OA	Decision twin	Clinical data, PROMs, AI	Predictive modeling	Shared decision-making	Randomized clinical trial
Hoyer et al.	2025	Arthroplasty / OA	Knee	Simulation-derived digital twin	Imaging, qMRI biomarkers	Imaging-based biomarker modeling	OA progression and knee replacement risk assessment	Cross-sectional cohort analysis
Suresh et al.		Spine	Thoracic spine	Monitoring twin	Surface scanning	Digital measurement twin	Deformity monitoring	Validation
Suresh et al.	2025	Spine	Paediatric spine	Monitoring twin	Surface scanning	Digital scoliometer DT	Telemedicine follow-up	Validation
Andres et al.	2025	Trauma	Long bones	Simulation twin	CT	FEA-based DT workflow	Revision planning	Case series
Di Matteo et al.	2024	Rehabilitation	Hand	Monitoring / hybrid twin	Wearables	Double DT framework	Rehab tracking	Feasibility
Frossard et al.	2022	Rehabilitation	Residuum	Monitoring twin	Sensors	Musculoskeletal DT	Prosthetic optimization	Validation
Quinn et al.	2023	Multidomain	Knee / MSK	Hybrid twin	Imaging, sensors	Control-oriented DT	Experimental control	Experimental
Saxby et al.	2023	Multidomain	MSK system	Hybrid twin	Multimodal	Neuromusculoskeletal DT	Conceptual framework	Conceptual

Footnote

Digital twin category reflects the functional classification proposed in the main manuscript and distinguishes static patient-specific simulations from monitoring-, decision-, and hybrid digital twin systems. Evidence level refers to the highest level of validation or clinical evaluation reported in the cited study.

Study Year	Orthopedic domain	Anatomical focus	Digital twin category	Core data inputs	Modeling approach	Intended clinical use	Evidence level

To będzie zgodne z main manuscript i dużo bezpieczniejsze.

Mój werdykt końcowy
